# Classifying the unclassifiable—a Delphi study to reach consensus on the fibrotic nature of diseases

**DOI:** 10.1093/qjmed/hcad050

**Published:** 2023-04-02

**Authors:** G M Massen, R J Allen, O C Leavy, N M Selby, G P Aithal, N Oliver, H Parfrey, L V Wain, G Jenkins, I Stewart, J K Quint

**Affiliations:** National Heart and Lung Institute, Imperial College London, London, W12 0BZ, UK; Department of Population Health Sciences, University of Leicester, Leicester, LE1 7RH, UK; NIHR Leicester Biomedical Research Centre, University of Leicester, Leicester, LE3 9QP, UK; Department of Population Health Sciences, University of Leicester, Leicester, LE1 7RH, UK; NIHR Leicester Biomedical Research Centre, University of Leicester, Leicester, LE3 9QP, UK; Department of Renal Medicine,, Derby Hospitals Foundation Trust, Derby, DE22 3NE,UK; NIHR Nottingham Digestive Diseases Biomedical Research Unit, University of Nottingham, Nottingham, NG7 2UH, UK; Department of Metabolism, Digestion and Reproduction, Imperial College London, London, SW7 2AZUK; Cambridge Interstitial Lung Disease Unit, Royal Papworth Hospital, Cambridge, CB2 0AY, UK; Department of Population Health Sciences, University of Leicester, Leicester, LE1 7RH, UK; NIHR Leicester Biomedical Research Centre, University of Leicester, Leicester, LE3 9QP, UK; National Heart and Lung Institute, Imperial College London, London, W12 0BZ, UK; National Heart and Lung Institute, Imperial College London, London, W12 0BZ, UK; National Heart and Lung Institute, Imperial College London, London, W12 0BZ, UK

## Abstract

**Background:**

Traditionally, clinical research has focused on individual fibrotic diseases or fibrosis in a particular organ. However, it is possible for people to have multiple fibrotic diseases. While multi-organ fibrosis may suggest shared pathogenic mechanisms, yet there is no consensus on what constitutes a fibrotic disease and therefore fibrotic multimorbidity.

**Aim:**

A Delphi study was performed to reach consensus on which diseases may be described as fibrotic.

**Methods:**

Participants were asked to rate a list of diseases, sub-grouped according to eight body regions, as ‘fibrotic manifestation always present’, ‘can develop fibrotic manifestations’, ‘associated with fibrotic manifestations’ or ‘not fibrotic nor associated’. Classifications of ‘fibrotic manifestation always present’ and ‘can develop fibrotic manifestations’ were merged and termed ‘fibrotic’. Clinical consensus was defined according to the interquartile range, having met a minimum number of responses. Clinical agreement was used for classification where diseases did not meet the minimum number of responses (required for consensus measure), were only classified if there was 100% consensus on disease classification.

**Results:**

After consulting experts, searching the literature and coding dictionaries, a total of 323 non-overlapping diseases which might be considered fibrotic were identified; 92 clinical specialists responded to the first round of the survey. Over three survey rounds, 240 diseases were categorized as fibrotic via clinical consensus and 25 additional diseases through clinical agreement.

**Conclusion:**

Using a robust methodology, an extensive list of diseases was classified. The findings lay the foundations for studies estimating the burden of fibrotic multimorbidity, as well as investigating shared mechanisms and therapies.

## Introduction

Fibrotic diseases are destructive conditions associated with ageing and metabolic abnormalities that occur in response to a range of known and unknown genetic and environmental factors. It is possible that fibrosis occurring in different organs may benefit from shared management strategies, reducing the burden of treatment through therapeutic rationalization, and early identification of secondary organ involvement. It has been proposed that organ fibrosis accounts for an estimated one-third of all deaths world-wide, although consensus of what constitutes fibrotic disease remains to be determined.

Diagnosis of fibrotic conditions is commonly made using combinations of radiological, and histopathological information. Whilst previous studies^1^ have outlined what diseases are fibrotic this has not been consensus based and was not considered to capture all fibrotic conditions. Due to current diagnostic pathways, clinical research currently focuses on individual fibrotic diseases and organ/system-specific pathologies. However, fibrotic conditions have an increased risk of co-morbidities. To address multi-morbid fibrotic disease, an interdisciplinary consortium plan to explore the mechanisms involved: DEfining MechanIsms Shared across mulTI-organ FIbrosis to prevent the development of long-term multimorbidity: DEMISTIFI-Multi Morbidity.

To understand whether there are shared pathogenic mechanisms within the multi-organ fibrotic disease, it is important to agree a definition of what constitutes a fibrotic disease in different organ systems. We therefore undertook a Delphi survey to identify where consensus does, or does not, exist regarding the clinical classification of fibrotic diseases in a range of organ systems.

## Methods

### Study design

A Delphi study with a minimum of two rounds was planned to obtain clinical consensus on the fibrotic classification of diseases across eight body regions. An anonymous survey was designed to minimize any professional hierarchy. All surveys were produced and distributed using a secure survey platform (Qualtrics.com).

Advice was sought from the steering committee of the DEMISTIFI-Multi Morbidity consortium, funded by a UKRI Strategic Priority award. An initial pilot survey was shared amongst both the steering committee and selected clinical experts who were representative of the body regions and medical subspecialties. The Delphi survey was subsequently refined according to pilot feedback.

### Identification of diseases

Lists of potentially fibrotic conditions were gathered in the literature, building upon Piera-Velazquez’s^2^ table of fibrotic diseases which was further updated in 2017 by Rosenbloom *et al.*^1^ Disease lists were subsequently expanded based on Systematised Nomenclature of Medicine Clinical Terms (SNOMED-CT) or International Classification of Disease 10th Revision (ICD-10) codes used in medical settings to record diagnoses. Any diseases which had not been included in the pilot or Round 1, which were subsequently suggested by respondents in free-text, were included in subsequent rounds.

To determine the diseases which could be included in the survey, the following databases were searched: SNOMED-CT, ICD-10 and PubMed literature using search terms (fibrosis OR fibrotic).

Diseases were subdivided into eight groups, namely diseases affecting primarily the: respiratory system, reproductive system, circulatory system, nervous system (including eyes), digestive system, urinary system, skin tissues and bones and systemic. By grouping diseases in this manner, participants only responded to diseases relative to their organ/system of expertise. All survey participants were asked to categorize systemic diseases.

### Definition of fibrotic classification

As no current clinical definition for fibrotic classification exists, the steering committee defined four groups for the definition of fibrotic disease classification. These definitions were introduced at the beginning of the survey and refined accordingly (Table 1).

**Table 1.** Definition of fibrotic classifications used in Delphi survey

	Explanation	Survey definition
Fibrotic manifestation always present	Diseases which always display fibrotic manifestations such as scarring and organ damage.	Diseases which are always fibrotic in nature.
Can develop fibrotic manifestations	Diseases which can display fibrotic manifestations, however not every patient with this diagnosis will develop fibrosis during the disease course.	Diseases which do not begin with fibrotic manifestation but can progress to fibrosis.
Associated with fibrotic manifestations	Diseases which do not display fibrotic manifestations at any stage. However, the disease is often comorbid with fibrotic conditions.	The disease itself is not fibrotic but is associated with other fibrotic manifestations.
Not fibrotic or associated	Diseases with no known link to fibrotic conditions or complications.	Diseases where there is no established link to a fibrotic condition or complication.

### Pilot survey

A pilot survey was released for the purpose of ensuring the final survey would gather the intended information (classification of diseases) and to ensure survey questions were clear and would achieve the survey aims as well as to provide information on survey length. The intention of the pilot was not to gather classifications of specific diseases. The Pilot was released to eight participants representing the eight predefined body regions. The pilot survey was also shared with three members (two non-clinicians and one clinician) of the DEMISTIFI consortium for review. A total of seven responses were received. Pilot members were asked at the end of the survey to rate the accessibility (was the survey easy to navigate and the survey length appropriate), comprehensiveness (were the list of diseases extensive and thorough) and interpretability (were questions clear or misleading) out of 100. The accessibility, comprehensiveness and interpretability of the survey were rated as 93%, 83% and 77%, respectively. As a result of the survey, the following changes were implemented: clearer definitions of the fibrotic nature classifications (to aid interpretability), lists split into groups of 10 for ease of viewing (to increase accessibility), demographic question and progress bar added. The pilot survey yielded three more diseases to be included in future survey; two in gastrointestinal (intestinal stricture, stricturing Crohn’s disease) and one in urinary system (renal tract fibrosis).

### Panel selection

The survey was aimed at clinicians and clinical researchers. The initial survey was distributed via email to 516 panel members, email addresses were gathered from professional websites (medical clinics or university) or from the corresponding author details of papers that had been published researching fibrotic diseases or mechanisms of fibrosis. Also, 19 charities were contacted, informed of the survey and asked to share it with their clinical advisory boards. Survey participants were asked to select the body regions for which they would feel comfortable classifying the nature of diseases. Multiple body regions could be selected. All participants were asked to classify the fibrotic nature of systemic diseases. At the end of Round 1, respondents were given a choice to opt-in to participate in further rounds of the survey by providing their email address for further survey distribution. All contact details were stored independently of the survey responses and were not linked.

## Consensus measure

To measure consensus, classifications of ‘fibrotic manifestation always present’ and ‘can develop fibrotic manifestations’ were merged and considered to indicate fibrosis. This was because fibrosis can be a progressive condition and therefore a distinction is not easy to make. The classifications were enumerated as follows: (1) not fibrotic or associated, (2) associated with fibrotic manifestations and (3) fibrotic manifestations always/sometimes present. Interquartile range (IQR) was calculated for each disease and if the IQR was equal to 0 then the disease could be said to have reached a consensus respective of IQR.^3^

Consensus was defined according to the interquartile range, having met a minimum number of participant responses (defined per body region), to yield stronger results where more responses are available but not to ignore results where less people responded to the organ. Respondents were not required to classify diseases that they were unfamiliar with, leading to varying response numbers per disease within the same body region. To reach a consensus on the classification of any given disease a minimum of five classification selections^4^ were required for diseases whose respective body region received ≤11 responses. Extrapolations of this defined a minimum criterion of 10 classification selections for diseases within body regions receiving between 12 and 20 responses and 15 classification selections for body regions receiving more than 20 responses.

## Agreement measure

For diseases that did not receive the minimum number of classification selections, an agreement measure was created. A disease had reached agreement if 100% of the respondents had classified the disease in the same way.

## Patient and public involvement

The DEMISTIFI consortium has a large PPIE network who contributed to design of the study.

## Results

### Survey responses

**Figure 1.**
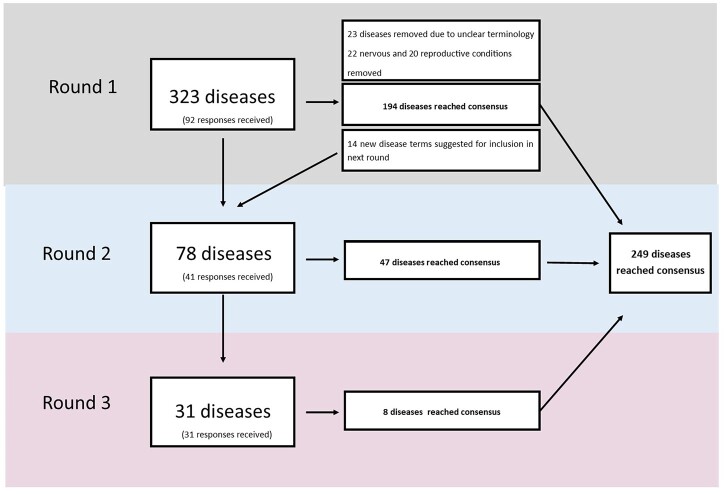
Flowchart of survey methodology.

In total, 516 individual survey invitations and 19 charity invitations were sent by email. The first round of the survey received 92 responses. As the charity invitations were intended to be cascaded to further recipients via their clinical advisory boards, it was not possible to determine response rate for Round 1. Of the responses, 77.2% were from participants in Europe, 19.6% from participants in the Americas, 1% in Southeast Asia and 2% from the Western Pacific. A total of 323 diseases were presented to survey participants to classify regarding their fibrotic nature. In Round 1, 194 diseases reached consensus on their fibrotic nature; five were defined as (1) ‘not fibrotic or associated’, the remaining 188 diseases were classified as (3) ‘fibrotic’. No diseases were categorized as (2) ‘associated with fibrotic manifestations’ (Figure 1).

Only 4 and 5 participants responding within neurological systems and reproductive systems respectively wished to be contacted in the next round this was too low for consensus to be reached and so diseases within these regions were excluded from further rounds (22 nervous and 20 reproductive). An additional 23 diseases were also removed from further surveys due to unclear terminology (e.g. diseases which had ‘with’ or ‘due to’ in their name).

At the end of Round 1, 61 participants opted-in to taking part in future survey rounds and a further 14 diseases were suggested for Round 2, providing 127 unclassified diseases.

A total of 78 diseases across six body regions were included in Round 2, which received a response rate of 67.2% (41/61). As a result of the second round, 47 of the diseases reached consensus, all of which were classified as (3) ‘fibrotic’ (Figure 1).

The final round included 31 diseases that required consensus classification and received 31 responses at a response rate of 50.8% (31/61). A total of eight diseases reached a consensus on their fibrotic nature; four of these diseases were classified as fibrotic (3) (Figure 1).

### Fibrotic classifications

**Figure 2.**
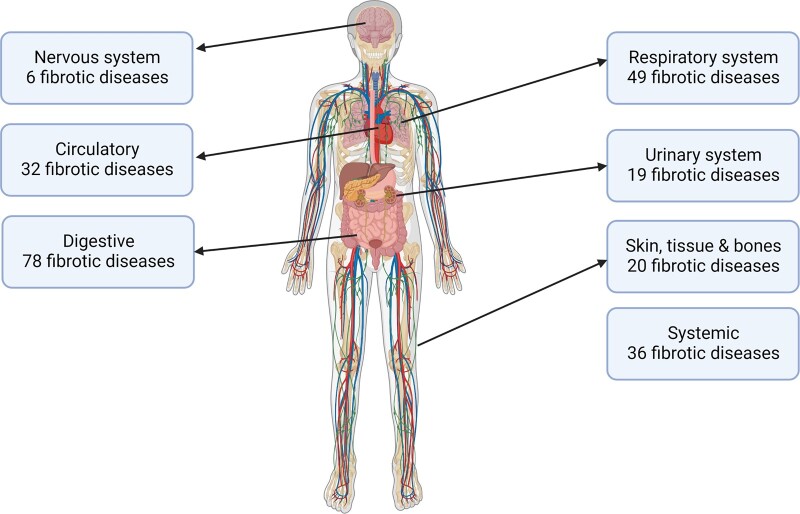
Number of diseases categorized as fibrotic per body region. Created with biorender.com.

**Figure 3.**
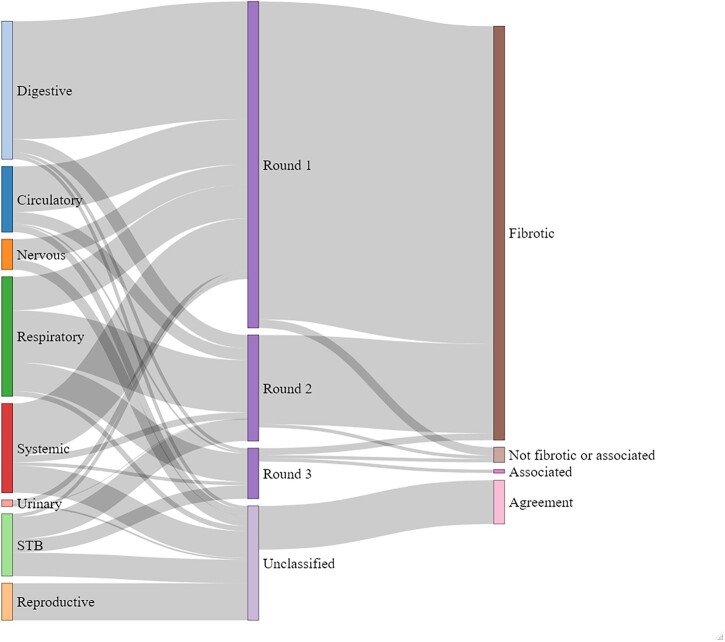
Sankey diagram displaying classifications per round. STB, skin, tissue and bone.

As a result of the survey, out of the 323 diseases included a total of 249 diseases were classified regarding their fibrotic nature. A total of 240 diseases were classified as fibrotic (Figure 2, Supplementary File S1). Two diseases reached consensus as ‘associated with fibrotic manifestations’ and seven diseases reached consensus as ‘neither fibrotic nor associated’. It was possible to classify 100% of the systemic diseases, 97.56% of the digestive diseases, 95.00% of the diseases of the urinary system, 96.23% of the respiratory diseases, 89.74% of the cardiac diseases and 60% of the diseases effecting the skeleton, skin and soft tissue diseases, 21.52% of nervous diseases (Figure 3).

### Fibrotic classifications via agreement

A total of 65 diseases were unclassified after three survey rounds. Clinical agreement was used in these instances to classify any diseases with 100% agreement on classification. Of the 65 diseases, 26 reached clinical agreement regarding their fibrotic nature, 25 of which were classified as fibrotic diseases (Figure 3, Supplementary File S2).

## Discussion

Overall, clinical consensus and agreement on the fibrotic nature of 275 diseases were attained. This included 265 diseases classified as having fibrotic manifestations always or sometimes present. Three survey rounds were required to reach clinical consensus/agreement on the fibrotic classification of 87.58% of the proposed diseases; demonstrating that the classification of the fibrotic nature of diseases is not straightforward or well understood.

Clinical consensus obtained through Delphi method resulted in validation of the fibrotic disease list presented by Rosenbloom *et al.*^1^ Of the 29 diseases in Rosenbloom’s list, 26 conditions were classified as being fibrotic in the first round of the survey and a further 2 (polycystic kidney disease and diffuse fasciitis) were classified as fibrotic in the second round of the survey (Table 2). Peyronie’s disease was not classified.

**Table 2.** Comparison of (Rosenbloom *et al*., 2017)^1^ disease list

Disease	Round	No. responses for ‘Fibrotic manifestation always present’	No. responses for ‘Can develop fibrotic manifestations’	No. responses for ‘Associated with fibrotic manifestations’	No. responses for ‘Not fibrotic or associated’
Systemic sclerosis	1	19	34	2	0
Multifocal fibrosclerosis	1	8	1	0	0
Nephrogenic systemic fibrosis	1	31	3	2	1
Sclerodermatous graft vs. host disease	1	12	24	2	3
Hypertension-associated cardiac fibrosis	1	9	0	0	0
Post-myocardial infarction	1	6	3	0	0
Chagas disease-induced myocardial fibrosis	1	5	1	0	0
Diabetic and hypertensive nephropathy	1	2	13	0	0
Urinary tract obstruction-induced myocardial fibrosis	1	11	2	1	0
Inflammatory/autoimmune-induced kidney fibrosis	1	10	4	0	0
Aristolochic acid nephropathy	1	4	6	0	1
Polycystic kidney disease	1	0	9	2	3
	2	0	7	0	2
Idiopathic pulmonary fibrosis	1	43	1	0	0
Silicosis	1	16	18	2	0
Asbestosis	1	29	12	1	0
Chemotherapeutic agent-induced pulmonary fibrosis					
Alcoholic and non-alcoholic liver fibrosis	1	16	8	2	0
Hepatitis C-induced liver fibrosis	1	20	1	2	0
Primary biliary cirrhosis	1	11	8	0	0
Parasite-induced liver fibrosis	1	12	4	1	0
Radiation-induced fibrosis	1	35	11	0	1
Bladder fibrosis	1	11	1	0	0
Intestinal fibrosis	1	17	2	2	1
Peritoneal sclerosis	1	24	12	0	1
Diffuse fasciitis	1	2	5	2	1
	2	1	3	1	0
Localized scleroderma	1	10	1	1	1
Dupytren’s disease	1	11	0	0	0
Peyronie’s disease	0	0	0	0	0
Myelofibrosis	1	10	1	1	0
Oral submucous fibrosis	1	13	3	0	1

All participants were asked to classify diseases into four definitions; however, on receipt of responses from Round 1, a primary analysis was performed based on a combination of two definitions (fibrotic manifestations always/sometimes present) in order to address consensus on the presence of fibrotic manifestations.

For the purposes of the DEMISTIFI consortium, diseases which did not reach clinical consensus or agreement but have ‘fibrosis’ within the name will be considered as fibrotic.

### Strengths and limitations

This study is the first of its kind looking to characterize diseases based on their fibrotic manifestation using clinical consensus and agreement measures. This global survey of clinicians has systematically classified an extensive list of conditions and provides a strong base for further fibrotic research, especially research looking to underpin links between fibrotic conditions. This too will be used to facilitate the calculation of regional and global fibrotic burden.

It was not possible to classify all diseases; however, it was possible to achieve either clinical consensus or clinical agreement on the classification of 275 of the diseases, of which 265 were classified as fibrotic.

Whilst the distribution of survey rounds was not restricted by country, it is possible that the opinions provided in the Delphi survey are not representative of clinical consensus globally. Similarly, responses may be limited to health professionals with resource capacity that may have facilitated optional participation. Despite this, the demographics of participants where provided indicated most respondents were either located in Europe or the Americas.

We have focused on a clinical definition of fibrosis, rather than consensus from pathologists who may have differing views on which diseases are fibrotic according to histopathology. Biopsy is not easily performed in all organs to assess fibrosis (lung, brain and heart) therefore there is an urgent need to classify diseases based on clinic-radiological assessment of fibrosis. This is a limitation but there is scope for future research to validate this extended list of fibrotic diseases by pathologists. Whilst we extend the list of fibrotic diseases based on clinical consensus from 29 to 265, it is evident that there are differences in opinion on fibrotic diseases warranting detailed investigations into mechanisms and co-occurrence.

## Conclusion

We provide a consensus-based classification of fibrotic diseases which extends previous classifications and provides the foundations for fibrotic multimorbidity studies to investigate shared mechanisms and therapies, as well as patient and clinical burdens. In conclusion, an extensive list of fibrotic diseases was classified according to clinical consensus using a robust methodology.

## Supplementary Material

hcad050_Supplementary_Data

## Aknowledgements

We are grateful to everyone who took part in survey development as well as responding to the survey, including Ralf Weiskirchen, Nazia Chaudhuri, Gavin Murphy, Gabriela C Tabaj, Claude Jourdan le Saux, Robert Scott, Diego Moriconi, Peter Boor, Bibek Gooptu, Timothy Kendall, Christopher Fry, Elisabeth Bendstrup, Dean Sheppard, Jesper Rømhild Davidsen, Scott Friedman, Kerri Johannson, John Greenland, Christine Fiddler, Eduardo Martin-Nares, Ole Hilberg, Alastair Moss, Guruprasad Aithal, Jonathan A Fallowfield, Joseph Jacob, Alberto Ortiz, Hilary Longhurst, Liz Lightstone, Vincent Cottin, Ivette Buendia-Roldan, Helen Parfrey, Giulio Marchesini.
