# Correction to: Classifying the unclassifiable—a Delphi study to reach consensus on the fibrotic nature of diseases

**DOI:** 10.1093/qjmed/hcad156

**Published:** 2023-07-01

This is a correction to: G M Massen and others, on behalf of the DEMISTIFI Consortium, Classifying the unclassifiable—a Delphi study to reach consensus on the fibrotic nature of diseases, *QJM: An International Journal of Medicine*, Volume 116, Issue 6, June 2023, Pages 429–435, https://doi.org/10.1093/qjmed/hcad050

In the originally published version of this manuscript, the **Acknowledgements** section was omitted. The text there should read: “We are grateful to everyone who took part in survey development as well as responding to the survey, including Ralf Weiskirchen, Nazia Chaudhuri, Gavin Murphy, Gabriela C Tabaj, Claude Jourdan le Saux, Robert Scott, Diego Moriconi, Peter Boor, Bibek Gooptu, Timothy Kendall, Christopher Fry, Elisabeth Bendstrup, Dean Sheppard, Jesper Rømhild Davidsen, Scott Friedman, Kerri Johannson, John Greenland, Christine Fiddler, Eduardo Martin-Nares, Ole Hilberg, Alastair Moss, Guruprasad Aithal, Jonathan A Fallowfield, Joseph Jacob, Alberto Ortiz, Hilary Longhurst, Liz Lightstone, Vincent Cottin, Ivette Buendia-Roldan, Helen Parfrey, Giulio Marchesini.”

This has been emended in the article.

