# Supplementary material for: Classifying the unclassifiable—a Delphi study to reach consensus on the fibrotic nature of diseases
Source: QJM. 2023 Apr 2;116(6):429–35. doi: 10.1093/qjmed/hcad050 (PMC10250078; doi:10.1093/qjmed/hcad050)
Supplement: hcad050_Supplementary_Data [file hcad050_supplementary_data.zip › hcad050_Supplementary_Data/Supplementary file 2.pdf]

Disease	R1 - Fibro	R1- Assoc	R1- None		R2 - Fibro	R2 - Assoc	R2 - None		R3 - Fibro	R3 - Assoc	R3 - None
**1. Aortic insufficiency**	7	2	1		3	2	0		3	3	0
**2. Obstructive hypertrophic cardiomyopathy**	6	1	1		3	3	0		2	2	0
**3. Restenosis**	5	1	1		5	2	0		3	3	0
**4. Vasculitis**					5	2	0		4	2	0
**5. Portal hypertension**	11	6	1		9	5	0		3	5	0
**6. Stenosis of anus & rectum**	12	3	2		9	3	0		5	2	0
**7. Bronchiolitis obliterans**	24	10	6		18	7	0		14	4	1
**8. Centrilobular emphysema**	11	9	21		17	6	0		4	5	9
**9. Epidermolysis bullosa dystrophica**					3	0	0		1	0	1
**10. Fibrosing poikiloderma ***	8	1	0		2	2	0		2	0	0
**11. Glial scar ***	6	0	1		4	0	0		4	0	0
**12. Lichen sclerosus ***	5	1	2		4	0	0		4	0	0
**13. Lipodermatosclerosis ***	7	1	0		4	0	0		2	0	0
**14. Stiff skin syndrome ***	8	0	0		3	0	0		3	0	0
**15. Subepidermal nodular fibrosis ***	9	0	0		3	0	0		3	0	0
**16. Reticulin fibrosis ***	3	0	0		3	0	0		2	0	0
**17. Duchenne muscular dystrophy ***	4	1	4		2	2	0		2	0	0
**18. Polymyalgia rheumatica ***	0	3	5		4	0	0		0	0	3
**19. Spinal cord injury ***					5	0	0		0	0	0
**20. Capsular fibrosis of temporomandibular joint ***	9	0	0		4	0	0		4	0	0
**21. Hypermobility syndrome**	2	0	5		4	0	0		1	0	2
**22. Atherosclerosis of renal artery**	8	3	0		3	3	0		2	4	0
**23. Age related macular degeneration**	10	0	4								
**24. Alzheimer's disease**	5	2	5								
**25. Amyotrophic Lateral Sclerosis**	6	2	3								
**26. Brain abscess**											

27. Congenital stenosis and stricture of lacrimal duct	5	1	1								
28. Corneal fibrosis *	9	0	0								
29. Diabetic retinopathy	1	0	1								
30. Diffuse subretinal fibrosis	1	0	0								
31. Epidural fibrosis *	8	0	0								
32. Fibrosis of retina *	8	0	0								
33. Glaucoma	4	1	5								
34. Multiple sclerosis	8	1	2								
35. Pituitary fibrosis *	8	0	0								
36. Preretinal fibrosis	0	0	0								
37. Restrive strabismus due to orbital fibrosis	0	0	0								
38. Retinal fibrosis	0	0	0								
39. Retinopathy of prematurity	5	2	2								
40. Subretinal fibrosis *	1	0	0								
41. Subretinal fibrosis and uveitis syndrome *	1	0	0								
42. Vitreous fibrosis *	9	0	0								
43. Traumatic brain injury											
44. Stroke											
45. Congestion-fibrosis syndrome (pelvic congestion syndrome)	1	0	1								
46. Endometrial fibrosis *	3	0	0								
47. Endometriosis *	3	0	0								
48. Fibrosis of cervix *	2	0	0								
49. Fibrosis of corpus cavernosum *	2	0	0								
50. Fibrosis of penis *	3	0	0								
51. Fibrosis of perineum *	2	0	0								
52. Fibrosis of scrotum *	2	0	0								
53. Fibrosis of seminal vesicle	1	1	0								
54. Fibrosis of spermatic cord	1	1	0								
55. Fibrosis of testis	1	1	0								
56. Fibrosis of tunica vaginalis	1	1	0								

**57. Fibrosis of uterus**	1	1	0								
**58. Fibrosis of vas deferens**	1	1	0								
**59. Leiomyoma of uterus**	2	0	1								
**60. Parametrial fibrosis**	1	1	0								
**61. Pelvic fibrosis**	1	1	0								
**62. Peyronie's disease**	2	1	0								
**63. Polycystic ovary syndrome**	1	0	2								
**64. Tubal fibrosis ***	2	0	0								
